# Metabolic fate of rambutan (*Nephelium lappaceum* L.) peel polyphenols following oral administration in rats

**DOI:** 10.1016/j.fochx.2026.103870

**Published:** 2026-04-15

**Authors:** Qiuming Liu, Wanmei Biao, Xingchun Duan, Yujie Zhong, Qingyu Ma, Liping Sun, Yongliang Zhuang

**Affiliations:** a Faculty of Food Science and Technology, Kunming University of Science and Technology, Kunming 650500, China; b School of Chemistry and Resources Engineering, Honghe University, Mengzi 661100, China; c Yunnan Technology Innovation Center of Woody Oil, Kunming 650201, China; d Yunnan Key Laboratory of Plateau Food Advanced Manufacturing, Kunming 650500, China

**Keywords:** Ellagitannins, Metabolism, Bioavailability, Pharmacokinetics, Urolithins

## Abstract

This study investigated the *in vivo* metabolic fate of rambutan peel polyphenols (RPPs) in rats. RPPs consisted primarily of ellagitannins, notably geraniin (341.24 mg/g dry weight). Following a single oral gavage of RPPs, 75 metabolites derived from ellagic acid, gallic acid, corilagin, and urolithins were identified. The potential metabolic pathways of RPPs included phase I metabolism (hydrolysis) and phase II metabolism (methylation, sulfation, and glucuronidation). Some prototype compounds (ellagic acid, ellagic acid hexuronide, and ellagic acid pentoside II) were detected, but with low systemic exposure and rapid elimination. Dimethyl ellagic acid glucuronide II showed high exposure and long residence. Gallic acid conjugates were rapidly excreted. The urolithin A glucuronide was identified as the primary microbially derived urolithin conjugate. Host-microbial co-metabolism critically enhances RPPs bioavailability, explaining their efficacy and supporting functional applications.

## Graphical abstract

**Figure:**
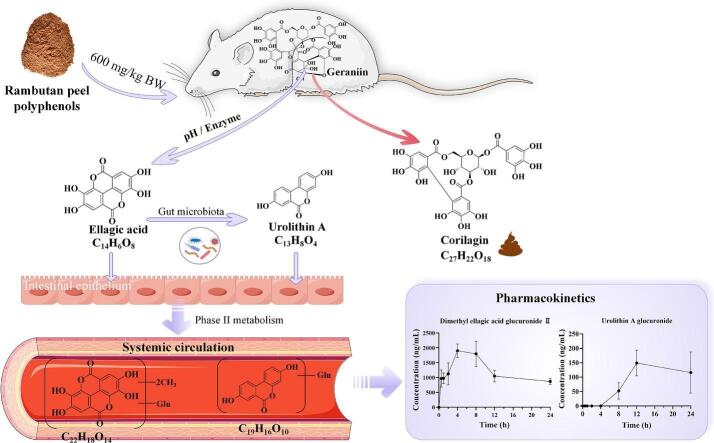

## 1 Introduction

Rambutan (*Nephelium lappaceum* L.) is a key tropical fruit, however, its peel—making up about 50% of the fruit mass—is largely discarded as waste, resulting in substantial resource loss and environmental issues (Rakariyatham, Zhou, Rakariyatham, & Shahidi, 2020). Recent studies have shown that rambutan peel extract was a potential source of valuable minerals and ellagitannins for industrial applications (Torgbo, Rugthaworn, Sukatta, & Sukyai, 2022), and exhibited antioxidant properties in food processing and storage systems (Mei et al., 2014; Phuong, Le, Nguyen, Van Camp, & Raes, 2019; Zou, Zhang, Dai, Liu, & Zang, 2025). These properties are largely attributed to its rich polyphenol content, primarily comprising geraniin, corilagin, and ellagic acid (Zhuang, Ma, Guo, & Sun, 2017a). Rambutan peel polyphenols (RPPs) have been demonstrated to possess excellent biological activities in *in vitro* and *in vivo* models, such as antioxidant (Albuquerque et al., 2023; Liu et al., 2025), anti-inflammatory (Li, Li, Hou, Zhuang, & Sun, 2018; Moorthy, Wie, Marino, & Palanisamy, 2022), antiglycation (Zhuang, Ma, Guo, & Sun, 2017b), antidiabetic (Liu, Ma, Li, Sun, & Zhuang, 2024; Ma, Guo, Sun, & Zhuang, 2017; Muhtadi, Primarianti, & Sujono, 2015), and anti-aging effects (Huang et al., 2025; Zhuang et al., 2017a). Geraniin, corilagin, and ellagic acid are considered the main contributors to the biological activities of RPPs (Liu, Sun, Zhong, Ma, & Zhuang, 2025). In fact, the beneficial effects of phenolic compounds are not solely attributable to their parent compounds, but are also influenced by metabolites and catabolites produced through digestion, microbial metabolism, and host biotransformation (Jakobek & Blesso, 2023).

Understanding the health benefits of polyphenols hinges on their oral bioavailability, defined by the efficiency of their absorption and systemic availability. Current research has revealed that RPPs face challenges such as poor water solubility and low stability (susceptible to temperature, pH, light, and microorganisms), which often result in low bioavailability (Liu, Sun, et al., 2025). Tan et al. found that after *in vitro* simulated gastrointestinal digestion of RPPs, the level of the main component geraniin decreased, while that of corilagin and ellagic acid increased after intestinal digestion (Tan, Ma, Li, Liu, & Zhuang, 2023). Due to glycosidic bond hydrolysis, quercetin and kaempferol increased after gastric digestion but decreased after intestinal digestion. Using a Caco-2 cell monolayer model, the study further revealed low permeability for geraniin and corilagin from the gastrointestinal digested RPPs, whereas ellagic acid, protocatechuic acid, and catechol showed relatively higher permeability (Tan et al., 2023). Mao et al. also found that, the absorption rate of ellagic acid in the intestine was higher than that of corilagin in a Caco-2 cell model (Mao et al., 2016). Although *in vitro* simulated gastrointestinal digestion led to a 27.30% decrease in total phenolic content, RPPs digestion product retained considerable antioxidant, α-glucosidase inhibitory, and pancreatic lipase inhibitory activities (Li et al., 2025). Moreover, gut microbiota also plays a significant role in influencing the absorption efficiency of RPPs. Phenolic compounds not absorbed in the gastrointestinal tract continue into the colon segment, where they are degraded and transformed by gut microbiota. Urolithins, as gut microbial metabolites of ellagitannins, are considered an important material basis for their health effects (Gandhi et al., 2022). In both *in vitro* colonic fermentation models and *in vivo* type 2 diabetic mouse models, geraniin and corilagin could be degraded by gut microbiota and ultimately transformed into a series of products including urolithins M5, D, C, A, and B (Li et al., 2025; Liu et al., 2024; Tan et al., 2023).

The biological activities of RPPs have been preliminarily confirmed; however, a significant knowledge gap exists regarding the paradox between their potent *in vivo* health effects and their extremely low bioavailability. It is therefore hypothesized that the *in vivo* metabolic conversion of RPPs, particularly by the gut microbiota, generates bioactive metabolites that serve as the true effectors. To test this hypothesis and systematically elucidate their metabolic fate, pharmacokinetic experiments in rats were conducted to identify the specific metabolites of RPPs, clarify the distinct metabolic roles of the host and gut microbiota. This work will deepen the scientific understanding of RPPs bioavailability and provide key evidence for promoting their transformation into functional foods or drug leads.

## 2 Materials and methods

### 2.1 Materials and reagents

Freshly purchased rambutans were cleaned to remove surface impurities. The rambutan peel powder was prepared by drying the peels at 60 °C, followed by grinding and sieving through a 60-mesh sieve. LC/MS grade acetonitrile was purchased from Merck (Darmstadt, Germany). Folin-Ciocalteu reagent, sodium carbonate, and LC/MS grade methanol were purchased from Shanghai Macklin Biochemical Technology Co., Ltd. (Shanghai, China). LC-MS grade formic acid was obtained from Honeywell (Shanghai, China). Catechin, corilagin, geraniin, gallic acid, syringic acid, kaempferol, protocatechuic acid, quercetin, rutin, and urolithin A were obtained from Shanghai Yuanye Bio-Technology Co., Ltd. (Shanghai, China).

### 2.2 Preparation of rambutan peel polyphenols

RPPs were prepared following the method described by Liu et al. (2024). Rambutan peel powder was mixed with 60% ethanol (*v*/v) and extracted ultrasonically at 240 W (5 min). Following centrifugation at 5000 rpm for 10 min, the supernatant was collected for the next step. The same extraction procedure was repeated for the residue. The combined supernatants were freeze-dried to obtain the crude extract, which was then further purified using NKA-9 macroporous resin in accordance with previous literature (Zhuang et al., 2017b), ultimately yielding RPPs. Briefly, the crude extract was loaded onto an NKA-9 resin column at a flow rate of 2 bed volume (BV)/h for dynamic adsorption. Subsequently, the resin column is rinsed with ultrapure water at a flow rate of 2 BV/h to remove residual water-soluble impurities. The column is then eluted with 60% ethanol at the same flow rate, and the eluted peak fractions are collected. Following freeze-drying, the RPPs are obtained.

### 2.3 Determination of total phenol content

Total phenolic content (TPC) of RPPs was quantified according to the Folin–Ciocalteu method described in previous studies (Tan et al., 2023). The sample (0.5 mL) was mixed with 2.5 mL of Folin−Ciocalteu reagent and allowed to stand for 5 min. Upon the subsequent addition of 2 mL of sodium carbonate solution (7.5%, w/v), the final mixture was kept in the dark for 60 min, and the absorbance was then measured at 765 nm. Gallic acid solutions (0–1 mg/mL) served as reference standards. The TPC value was showed as milligram gallic acid equivalents per gram of dry weight sample (mg GAE/g DW).

### 2.4 Animal experiments

Male Sprague-Dawley rats (6 weeks old, 200 ± 10 g) were purchased from SPF Biotechnology Co., Ltd. (Beijing, China, License No.: SCXK (Jing) 2024-0001). Rats were housed under a 12 h light/dark cycle (temperature 22 ± 2 °C, humidity 55% ± 5%) with free access to food and water, and acclimatized for 1 week. All animal experiments were conducted in strict accordance with Laboratory Animal Management in China, under the guidance of Laboratory Animal Care and Use, and were approved by the Experimental Animal Ethics Committee of Kunming University of Science and Technology (Approval No.: PZWH-KUST-202507010051-11).

In the experiment, 54 rats were randomly divided into blood concentration group (*n* = 48) and urine and feces excretion group (*n* = 6) according to the random number table. After a 12 h fast, rats received an oral administration of RPPs (600 mg/kg BW). The selection of the intragastric dose was based on our previous study, which demonstrated that, in subacute toxicity studies, SD rats showed no signs of toxicity even at RPPs doses as high as 625 mg/kg BW (Li, Zhuang, Tian, & Sun, 2020). Following oral administration, blood samples were collected from the abdominal aorta of 48 rats at eight time points (0, 0.5, 1, 2, 4, 8, 12, and 24 h), with 6 rats per time point and each rat sampled only once. The sample size was determined on the basis of our preliminary experiments and previous study (Yuan et al., 2023). Following collection, the blood was set aside at room temperature for 30 min and subsequently centrifuged (3500 rpm, 15 min) to separate the serum. Urine and feces were collected from 6 rats in metabolic cages before and 24 h after oral gavage of RPPs. All samples were immediately frozen at −80 °C until analysis. After the experiment, all the rats were anaesthetized by intraperitoneal injection of 1% sodium pentobarbital (40 mg/kg BW) and then euthanized by cervical dislocation.

### 2.5 Extraction and determination of rambutan peel polyphenol metabolites

#### 2.5.1 Extraction of rambutan peel polyphenol metabolites

Metabolites from biological samples were extracted based on a previously reported protocol (Zhang et al., 2023) with modifications. Serum (300 μL) and fecal samples (200 mg) were extracted by mixing with a pre-cooled methanol/acetonitrile/water (2:1:1, v/v) mixture. Fecal samples were further treated by low-temperature ultrasonication for 30 min. The mixture was subsequently centrifuged at 13,000 rpm for 15 min (4 °C). The collected supernatant was dried under a stream of nitrogen, and the resulting residue was redissolved in an acetonitrile/water (1:1, v/v) solution. For fecal samples, the reconstituted solution was centrifuged again at 13,000 rpm and 4 °C for 15 min. Finally, the supernatant from all samples was filtered through a 0.22 μm organic membrane filter prior to metabolite analysis. Due to the complex matrix of urine samples, solid-phase extraction was used for purification and enrichment, based on prior research (Cladis et al., 2021) and with minor modifications. Oasis PRiME HLB extraction cartridges (Waters Corporation, MA, USA) were used. The procedure included loading, washing with 5% methanol to remove polar interferents, and eluting with methanol. The resulting eluate was then dried under nitrogen, reconstituted, and filtered similarly to the other samples for metabolites analysis.

#### 2.5.2 Determination of rambutan peel polyphenols and their metabolites by UPLC-MS/MS

The measurement of RPPs and their metabolites was performed according to a previously established method (Liu, Ma, et al., 2025). Liquid chromatographic analysis was carried out on an Ultimate 3000 series Ultra Performance Liquid Chromatography (UPLC) system (Thermo Fisher Scientific, MA, USA). Separation was performed using a Poroshell 120 EC-C18 column (2.1 mm × 100 mm, 1.9 μm) maintained at 35 °C. The injection volume was 2 μL, with a mobile phase flow rate of 0.2 mL/min. The mobile phase consisted of 0.1% formic acid in water (solvent A) and acetonitrile (solvent B) using the following gradient: 5% B (0–1 min), 15% B (1–5 min), 38% B (5–10 min), 65% B (10–15 min), 80% B (15–18 min), and 100% B (18–22 min).

Mass spectrometry was performed on a Q Exactive hybrid quadrupole-Orbitrap mass spectrometer (Thermo Fisher Scientific, Bremen, Germany) with a negative electrospray ionization source. The settings included an ion source temperature of 350 °C, a spray voltage of 3.5 kV, a capillary temperature of 320 °C, and a scan range of *m*/*z* 100–1000. Data acquisition involved full MS scans at 70,000 resolution and MS^2^ scans at 35,000 resolution employing stepped collision energies of 10, 30, and 50 eV.

Data acquisition and processing were performed using XCalibur software (Thermo Fisher Scientific, MA, USA). Metabolite identification was achieved by matching the accurate molecular masses and MS^2^ fragmentation patterns against literature data and the Human Metabolome Database. The mass error during polyphenol identification is less than 5 ppm. To quantify phenolic metabolites in rats, the baseline levels at the 0 h time point must be subtracted from the measured concentrations at each subsequent time point. Quantification of phenolic compounds was conducted using external calibration curves (0–3.13 μg/mL) of authentic standards, including catechin, corilagin, geraniin, gallic acid, syringic acid, kaempferol, protocatechuic acid, quercetin, rutin, and urolithin A. In the absence of authentic standards, semi-quantitative analysis was carried out based on calibration curves established for structurally similar compounds.

### 2.6 Pharmacokinetic parameters of rambutan peel polyphenol metabolites and data analysis

The C_max_ was designated as the maximum serum concentration of RPPs metabolites measured during the 24 h period after administration, while T_max_ was recorded as the time point to achieve C_max_. The area under the curve (AUC_0__–__24_) represents the area enclosed by the serum concentration-time curve from 0 to 24 h, reflecting the systemic exposure to RPPs. The half-life (T_1/2_) indicates the time required for the plasma concentration of a metabolite to decrease by half. The mean residence time (MRT_0__–__24_) of the metabolites during the 0–24 h period was also determined. All pharmacokinetic parameters were calculated using non-compartmental analysis with Phoenix WinNonlin software (version 8.3).

## 3 Results

### 3.1 Rambutan peel polyphenols

The TPC value of RPPs was 664.32 mg GAE/g DW, and the phenolic compounds identified and quantified are summarized in Table 1. The phenolic profile of RPPs comprised 33 identified compounds, including 6 phenolic acids, 5 flavonols, 5 flavanols, 1 flavone, 6 ellagic acid conjugates and its metabolites, and 10 hydrolyzable tannins. Quantitative analysis revealed that hydrolyzable tannins were the most abundant components in RPPs, among which geraniin was the predominant constituent at 341.24 mg/g dry weight (DW). Other important tannins, such as corilagin (7.61 mg/g DW), HHDP-digalloylglucose I (8.30 mg/g DW), and various galloyl glucoses (tri-galloyl-hexoside, digalloyl-hexoside), also contributed significantly. Ellagic acid and its derivatives represented another key component class in RPPs. Notably high levels were found for ellagic acid (28.48 mg/g DW) and, in particular, several of its glycosidic derivatives ellagic acid pentoside II (21.70 mg/g DW), ellagic acid xyloside dimer (10.18 mg/g DW), and ellagic acid hexuronide (6.11 mg/g DW). Among the phenolic acids, syringic acid was the most abundant (36.69 mg/g DW), with its content substantially exceeding that of gallic acid (2.24 mg/g DW), protocatechuic acid (1.58 mg/g DW), and brevifolincarboxylic acid I (1.61 mg/g DW). Although RPPs contained a diverse profile of flavonoids (*e.g.*, flavonols, flavanols, and flavones), these compounds were only detected at low levels.

**Table 1:** Qualitative and quantitative analysis of phenolic compounds in rambutan peel polyphenols (*n* = 3).

Phenolic compound	Chemical formular	RT (min)	[M-H]^−^/(*m*/*z*)	MS^2^/(m/z)	Concentration (mg/g extract)
Phenolic acids					
4-Hydroxylbenzoic acid	C_7_H_6_O_3_	1.28	137.0236	65.0385, 93.0333	0.15 ± 0.00
Gallic acid	C_7_H_6_O_5_	2.22	169.0137	124.0157, 125.0235	2.24 ± 0.40
Protocatechuic acid	C_7_H_6_O_4_	4.22	153.0187	65.0022, 91.0178, 109.0285, 108.0207	1.58 ± 0.14
Brevifolincarboxylic acid I	C_13_H_8_O_8_	7.45	291.0153	219.0299, 247.0248, 291.0153	1.61 ± 0.39
Brevifolincarboxylic acid II	C_13_H_8_O_8_	8.00	291.0152	219.0299, 247.0248, 291.0153	0.52 ± 0.05
Syringic acid	C_9_H_10_O_5_	9.37	197.045	89.0020, 95.0126, 123.0077, 53.046, 160.8414, 197.0456	36.69 ± 1.34

Flavonols					
Quercetin 3-O-β-glucoside	C_21_H_20_O_12_	9.91	463.0898	151.0029, 178.9981, 255.0302,271.0257, 300.0285, 301.0351	0.98 ± 0.05
Kaempferol rutinoside I	C_27_H_30_O_15_	10.24	593.1534	284.0335, 285.0412	0.58 ± 0.00
Quercetin 3-O-β-rhamnoside	C_21_H_20_O_11_	10.59	447.0948	151.0028, 255.0303, 271.0254, 300.0282, 301.0357	0.70 ± 0.05
Kaempferol rhamnoside II	C_21_H_20_O_10_	11.23	431.0995	227.0351, 255.0303, 284.0332, 285.0409	0.74 ± 0.54
Quercetin	C_15_H_10_O_7_	12.39	301.0352	197.8074, 301.0356	0.05 ± 0.00

Flavanols					
Procyanidin dimer I	C_30_H_26_O_12_	7.84	577.1375	125.0236, 245.0824, 289.0728, 407.0785, 425.0894	1.20 ± 0.74
Cyanidin-3-O-glalctoside	C_21_H_22_O_11_	8.00	449.11	287.0556	1.20 ± 0.04
Catechin	C_15_H_14_O_6_	8.37	289.0725	97.0284, 109.0283, 123.0442, 125.0235	1.67 ± 0.00
Cyanidin-4-O-glalctoside	C_21_H_22_O_11_	8.53	449.1099	287.0566	1.70 ± 0.01
Prodelphinidin dimer	C_30_H_26_O_14_	11.61	609.127	300.0282, 301.0355, 463.0894	2.84 ± 0.14

Flavones					
Rutin	C_27_H_30_O_16_	9.66	609.1478	300.0282	0.59 ± 0.01

Ellagic acid conjugates and its metabolites					
Ellagic acid hexuronide	C_20_H_14_O_14_	8.46	477.0317	125.0234, 169.0136, 300.9996	6.11 ± 0.18
Ellagic acid pentoside I	C_19_H_14_O_12_	8.83	433.0424	299.9918, 300.9995	5.17 ± 0.19
Decarboxyellagic acid	C_13_H_8_O_7_	8.93	275.0203	229.0143, 275.0203	2.76 ± 0.23
Ellagic acid pentoside II	C_19_H_14_O_12_	9.31	433.0423	299.9918, 300.9995	21.70 ± 0.10
Ellagic acid xyloside dimer	C_20_H_36_O_37_	9.34	867.0923	433.0421, 434.0456	10.18 ± 0.13
Ellagic acid	C_14_H_6_O_8_	9.81	300.9997	145.0287, 185.0240, 201.0195, 245.0094, 283.9970, 300.9996	28.48 ± 1.98

Hydrolyzable tannins					
Galloylhexose I	C_13_H_16_O_10_	1.75	331.0683	169.0133	0.18 ± 0.01
Galloylhexose II	C_13_H_16_O_10_	2.25	331.0684	169.0132	0.20 ± 0.00
Valoneic acid dilactone	C_21_H_10_O_13_	5.26	469.0058	300.9977, 397.0214, 407.0052, 425.0160, 469.0058	1.01 ± 0.07
Digalloyl-hexoside	C_20_H_20_O_14_	6.83	483.0798	169.0139, 331.0688	3.37 ± 0.19
Corilagin	C_27_H_22_O_18_	8.29	633.0754	275.0204, 300.9997, 463.0528	7.61 ± 0.13
Geraniin	C_41_H_28_O_27_	8.35	951.0773	273.0047, 300.9997, 933.0662	341.24 ± 2.39
Tri-galloyl-hexoside	C_27_H_24_O_18_	8.65	635.0914	465.0680, 483.0788	5.69 ± 0.59
Galloyl-bis-HHDP-glucose	C_42_H_30_O_25_	8.78	935.0863	169.0135, 300.9996, 463.0527, 935.0817	2.51 ± 0.02
HHDP-digalloylglucose I	C_34_H_26_O_22_	9.48	785.0866	249.0410, 275.0205, 300.9997, 615.0645	8.30 ± 0.14
Tetragalloy glucose	C_27_H_32_O_27_	9.64	787.1018	465.0680, 617.0797, 787.1013	6.24 ± 0.45

### 3.2 Rambutan peel polyphenol metabolites in rat serum

In this study, SD rats were administered RPPs (600 mg/kg BW) *via* gavage to investigate its absorption and metabolic characteristics. The ellagitannin metabolites of RPPs in rats (serum, urine, feces) are listed in Table 2. A total of 75 metabolites were identified, including 27 ellagic acid and conjugates, 24 gallic acid and conjugates, 4 corilagin and conjugates, and 20 urolithins and conjugates. A number of ellagic acid derivatives, including ellagic acid, ellagic acid pentoside II, methyl ellagic acid I, dimethyl ellagic acid IV, and dimethyl ellagic acid V, were identified across the serum, urine, and fecal samples. Several RPPs prototype compounds, such as ellagic acid, ellagic acid pentoside Ι, ellagic acid pentoside II, and ellagic acid hexuronide, were also detected in rat serum. Urolithins (M5, D, A, C) and their conjugates were detected in different biological samples analyzed. Specifically, urolithin A and C along with their conjugates were predominantly present in urine and serum, whereas urolithin M5 and D and their conjugates were primarily recovered from feces. The detection of gallic acid and gallotannins (galloylhexose and digalloyl-hexoside) in the feces points to the hydrolysis of geraniin or galloyl glucoses as their source (Li, Sun, & Zhuang, 2021). Meanwhile, the presence of other gallic acid conjugates mainly in serum and urine suggests their systemic absorption and subsequent circulation. Similarly, corilagin and its related metabolites were only detected in feces.

**Table 2:** Metabolites of rambutan peel polyphenols in rats (n = 6 per time point).

No.	Serum metabolites	Formular	t_R_ (min)	[M-H]- (m/z)	MS^2^	Source
Ellagic acid and its conjugates						
1	Ellagic acid⁎	C_14_H_6_O_8_	9.85	300.9985	169.0131, 201.0185, 229.0142, 257.0087, 283.9964	S, U, F
2	Ellagic acid pentoside I⁎	C_19_H_14_O_12_	8.89	433.0431	299.9908, 300.9985	S, F
3	Ellagic acid pentoside II⁎	C_19_H_14_O_12_	9.34	433.0424	299.9911, 300.9982	S, U, F
4	Ellagic acid hexuronide⁎	C_20_H_14_O_14_	8.38	477.0303	300.9987, 477.0305	S
5	Methyl ellagic acid I	C_15_H_8_O_8_	11.28	315.0152	270.9871, 299.9911, 315.0149	S, U, F
6	Methyl ellagic acid II	C_15_H_8_O_8_	12.83	315.0147	270.9872, 299.9915, 315.0151	U
7	Methyl ellagic acid III	C_15_H_8_O_8_	13.53	315.0141	270.9868, 299.9913, 315.0149	S, U
8	Dimethyl ellagic acid I	C_16_H_10_O_8_	10.22	329.0297	270.9880, 298.9834, 314.0069	S, U
9	Dimethyl ellagic acid II	C_16_H_10_O_8_	11.24	329.0302	270.9878, 298.9830, 314.0066	U
10	Dimethyl ellagic acid III	C_16_H_10_O_8_	12.23	329.0297	270.9884, 298.9835, 314.0068	S, U
11	Dimethyl ellagic acid IV	C_16_H_10_O_8_	12.57	329.0292	270.9879, 298.9840, 314.0061	S, U, F
12	Dimethyl ellagic acid V	C_16_H_10_O_8_	12.86	329.0291	270.9885, 298.9837, 314.0066	S, U, F
13	Methyl ellagic acid sulfate I	C_15_H_8_O_11_S	10.11	394.9711	299.9913, 315.0144	U
14	Methyl ellagic acid sulfate II	C_15_H_8_O_11_S	10.75	394.9709	299.9909, 315.0138	S, U
15	Methyl ellagic acid sulfate III	C_15_H_8_O_11_S	11.15	394.9708	299.9912, 315.0142	S, U
16	Dimethyl ellagic acid sulfate I	C_16_H_10_O_11_S	10.40	408.9865	298.9831, 314.0072, 329.0032	S, U
17	Dimethyl ellagic acid sulfate II	C_16_H_10_O_11_S	10.82	408.9874	298.9830, 314.0071, 329.0034	S
18	Dimethyl ellagic acid sulfate III	C_16_H_10_O_11_S	11.04	408.9866	298.9829, 314.0071, 329.0031	S, U
19	Methyl ellagic acid glucuronide I	C_21_H_16_O_14_	8.35	491.0442	229.0149, 257.0105, 299.9919, 300.9994, 315.0142, 475.0162	U
20	Methyl ellagic acid glucuronide II	C_21_H_16_O_14_	8.97	491.0463	229.0138, 257.0103, 299.9915, 300.9999, 315.0144, 475.0158	S, U
21	Methyl ellagic acid glucuronide III	C_21_H_16_O_14_	9.66	491.0469	229.0145, 257.0108, 299.9916, 300.9990, 315.0138, 475.0166	S, U, F
22	Dimethyl ellagic acid glucuronide I	C_22_H_18_O_14_	9.84	505.0627	298.9835, 314.0068, 329.0298	U
23	Dimethyl ellagic acid glucuronide II	C_22_H_18_O_14_	10.14	505.0620	298.9829, 314.0061, 329.0300	S, U
24	Methyl ellagic acid diglucuronide I	C_27_H_24_O_20_	6.83	667.0789	299.9918, 315.0171	S
25	Methyl ellagic acid diglucuronide II	C_27_H_24_O_20_	7.05	667.0779	299.9930, 315.0175	S
26	Methyl ellagic acid diglucuronide III	C_27_H_24_O_20_	7.65	667.0784	299.9915, 315.0165	S
27	Methyl ellagic acid diglucuronide IV	C_27_H_24_O_20_	8.12	667.0778	299.9919, 315.0163	S

Gallic acid and its conjugates						
28	Gallic acid⁎	C_7_H_6_O_5_	2.36	169.0124	124.0157, 125.0235, 169.0134	U, F
29	Methyl gallic acid I	C_8_H_8_O_5_	5.64	183.0305	123.0074, 124.0146, 125.0174, 168.0061	S, U
30	Methyl gallic acid II	C_8_H_8_O_5_	5.90	183.0299	123.0070, 124.0150, 125.0171	S, U
31	Gallic acid sulfate I	C_7_H_6_O_8_S	2.64	248.9715	125.0235, 169.0131	S, U
32	Gallic acid sulfate II	C_7_H_6_O_8_S	2.95	248.9711	169.0132	S, U
33	Methyl gallic acid sulfate I	C_8_H_8_O_8_S	3.36	262.9866	124.0156, 137.0605, 168.0049, 183.0299	S
34	Methyl gallic acid sulfate II	C_8_H_8_O_8_S	4.08	262.9862	124.0155, 137.0592, 168.0047, 183.0291	S, U
35	Methyl gallic acid sulfate III	C_8_H_8_O_8_S	8.54	262.9867	124.0145, 168.0056, 183.0289	U
36	Dimethyl gallic acid sulfate I	C_9_H_10_O_8_S	7.30	277.0019	125.0235, 169.0139	S
37	Dimethyl gallic acid sulfate II	C_9_H_10_O_8_S	8.86	277.0021	125.0238, 169.0132	S, U
38	Dimethyl gallic acid sulfate III	C_9_H_10_O_8_S	10.59	277.0019	125.0242, 169.0138	S, U
39	Gallic acid glucuronide I	C_13_H_14_O_11_	2.21	345.0466	169.0139	S, U
40	Gallic acid glucuronide II	C_13_H_14_O_11_	2.90	345.0463	169.0137	S, U
41	Methyl gallic acid glucuronide I	C_14_H_16_O_11_	2.03	359.0612	124.0148, 168.0071, 183.0298	S
42	Methyl gallic acid glucuronide II	C_14_H_16_O_11_	2.83	359.0616	124.0141, 168.0068, 183.0293	S, U
43	Methyl gallic acid glucuronide III	C_14_H_16_O_11_	3.29	359.0621	124.0154, 168.0069, 183.0289	S, U
44	Methyl brevifolincarboxylate	C_14_H_10_O_8_	9.10	305.0298	217.0141, 245.0082, 273.0061	S, U, F
45	Ethyl brevifolincarboxylate	C_15_H_12_O_8_	10.55	319.0459	273.0036, 245.0085, 217.0141	F
46	Galloylhexose I	C_13_H_16_O_10_	1.47	331.0663	111.0069, 123.0071, 125.0222, 169.0129	U, F
47	Galloylhexose II	C_13_H_16_O_10_	2.14	331.0656	111.0069, 123.0069, 125.0225, 169.0126	U, F
48	Galloylhexose III	C_13_H_16_O_10_	2.47	331.0653	111.0069, 123.0065, 125.0231, 169.0125	U
49	Digalloyl-hexoside I	C_20_H_20_O_14_	5.86	483.0798	125.0228, 169.0135, 331.0691	U
50	Digalloyl-hexoside II	C_20_H_20_O_14_	6.42	483.0789	125.0231, 169.0137, 331.0688	U, F
51	Digalloyl-hexoside III	C_20_H_20_O_14_	7.05	483.0794	125.0225, 169.0139, 331.0694	U

Corilagin and its conjugates						
52	Corilagin⁎	C_27_H_22_O_18_	8.42	633.0754	275.0204, 300.9997, 463.0528	F
53	Hydrolysate of corilagin	C_20_H_18_O_14_	1.48	481.062	275.0195, 300.9996	F
54	Methylation of corilagin	C_28_H_24_O_18_	9.52	647.0908	463.0523, 300.9984	F
55	Dimethyl of corilagin	C_29_H_26_O_18_	10.47	661.1043	477.0715, 315.0139, 289.0356	F

Urolithins and their metabolites						
56	Urolithin A	C_13_H_8_O_4_	12.18	227.0346	149.0963, 171.0452, 182.9539, 198.0321, 227.0344	U
57	Urolithin A glucuronide	C_19_H_16_O_10_	9.34	403.0675	113.0226, 227.0335	S, U
58	Urolithin A sulfate	C_13_H_8_O_7_S	10.25	306.9915	146.9600, 182.9870, 227.0345	S, U
59	Urolithin A glucuronide and sulfate	C_19_H_16_O_13_S	7.71	483.0239	227.0346, 403.0664	S
60	Urolithin C	C_13_H_8_O_5_	10.77	243.0299	199.0389, 187.0384, 171.0435	F
61	Urolithin C sulfate	C_13_H_8_O_8_S	10.07	322.9848	243.0246	U
62	Urolithin C glucuronide I	C_19_H_16_O_11_	9.26	419.0611	175.0238, 187.0423, 214.9995, 243.0294	S, U
63	Urolithin C glucuronide II	C_19_H_16_O_11_	10.05	419.0614	175.0240, 187.0426, 214.9997, 243.0296	S
64	Urolithin C diglucuronide I	C_25_H_24_O_17_	7.67	595.0928	243.0240, 419.0610 K	S
65	Urolithin C diglucuronide II	C_25_H_24_O_17_	7.88	595.0934	243.0245, 419.0614	S
66	Methyl urolithin C glucuronide I	C_20_H_18_O_11_	9.87	433.0775	243.0248, 257.0481	S, U
67	Methyl urolithin C glucuronide II	C_20_H_18_O_11_	10.42	433.0787	243.0256, 257.0485	S, U
68	Urolithin D	C_13_H_8_O_6_	10.37	259.0248	213.0178, 242.0204	F
69	Methyl urolithin D glucuronide	C_21_H_22_O_11_	10.19	449.0702	259.0606, 273.0409	S
70	Dimethyl urolithin D sulfate I	C_15_H_12_O_9_S	8.57	367.0124	259.0604, 287.0564	U
71	Dimethyl urolithin D sulfate II	C_15_H_12_O_9_S	8.91	367.0129	259.0593, 287.0575	S, U
72	Dimethyl urolithin D sulfate III	C_15_H_12_O_9_S	9.47	367.0131	259.0610, 287.0568	U
73	Dimethyl urolithin D sulfate IV	C_15_H_12_O_9_S	11.04	367.0124	259.0602, 287.0572	F
74	Urolithin M5	C_13_H_8_O_7_	9.07	275.0197	203.0330, 229.0126, 257.0076	F
75	Urolithin M5 glucuronide I	C_19_H_16_O_13_	6.65	451.0491	275.0195	F

⁎ Metabolites present in both rambutan peel polyphenols and rat metabolites. S: serum; U: urine; F: fecal.

### 3.3 Pharmacokinetic parameters of rambutan peel polyphenol metabolites

**Fig. 1:**
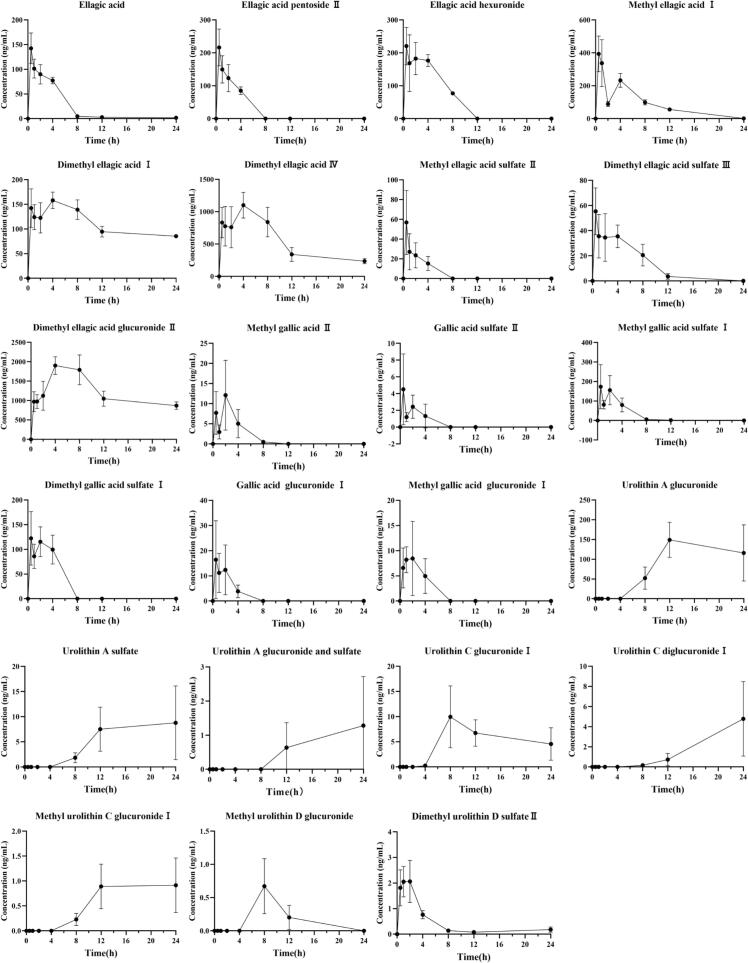
Time-dependent concentration curve of ellagitannins metabolites in rat serum.

The pharmacokinetic profiles of the 23 relatively abundant serum metabolites are summarized in Fig. 1 (concentration-time curves) and Table 3 (pharmacokinetic parameters). These metabolites include 9 ellagic acid and conjugates, 6 gallic acid and conjugates, and 8 urolithin conjugates. A bimodal pattern was observed in the concentration-time curves for multiple metabolites, indicating potential enterohepatic circulation (Fig. 1). These included ellagic acid hexuronide, methyl ellagic acid I, dimethyl ellagic acid I, dimethyl ellagic acid IV, dimethyl ellagic acid sulfate III, methyl gallic acid II, gallic acid sulfate II, methyl gallic acid sulfate I, dimethyl gallic acid sulfate I, and gallic acid glucuronide I. The prototype compounds ellagic acid, ellagic acid pentoside II, and ellagic acid hexuronide reached peak serum concentrations (C_max_ of 145.55, 224.05, and 252.04 ng/mL, respectively) around 1 h (T_max_), characterizing them as early-phase metabolites with relatively fast absorption. Furthermore, their concentrations fell below the detection limit after 8 h, indicating rapid metabolism. Among the ellagitannin metabolites, dimethyl ellagic acid glucuronide II was the most abundant in serum (C_max_ = 2005.41 ng/mL, AUC_0__–__24_ = 29,395.83 h·ng/mL) and exhibited the longest duration of action (MRT_0__–__24_ = 10.56 h, T_1/2_ = 27.80 h). It was followed by dimethyl ellagic acid IV (C_max_ = 1232.82 ng/mL, AUC_0__–__24_ = 12,937.71 h·ng/mL, which demonstrated a faster absorption (T_max_ = 2.58 h) and a sustained presence (MRT_0__–__24_ = 8.48 h). Both dimethyl ellagic acid I and IV exhibited pharmacokinetic profiles characterized by sustained exposure. Dimethyl ellagic acid I showed a high AUC_0__–__24_ (2648.96 h·ng/mL), long T_1/2_ (26.23 h), and MRT_0__–__24_ (10.66 h), while dimethyl ellagic acid IV displayed a similar pattern, indicating that both compounds, despite slightly slow absorption, provide a long-lasting effect. Several other ellagic acid conjugates exhibited low systemic exposure and rapid clearance. Specifically, methyl ellagic acid I, methyl ellagic acid sulfate II, and dimethyl ellagic acid sulfate III showed low C_max_ (431.06, 59.08, and 61.09 ng/mL, respectively), early T_max_ (0.67, 0.75, and 1.33 h, respectively), and short MRT_0__–__24_ (5.70, 1.70, and 4.33 h, respectively).

**Table 3:** Pharmacokinetic parameters of rambutan peel ellagitannins metabolites in rat serum.

Metabolite	T_1/2_ (h)	T_max_ (h)	C_max_ (ng/mL)	AUC_0-t_ (h*ng/mL)	MRT_0-t_ (h)
Ellagic acid	5.10 ± 1.96	0.75 ± 0.61	145.55 ± 27.33	567.03 ± 54.03	3.53 ± 0.31
Ellagic acid pentoside II	4.57 ± 2.09	0.75 ± 0.61	224.05 ± 47.77	489.79 ± 76.79	1.79 ± 0.11
Ellagic acid hexuronide	5.96 ± 2.18	1.42 ± 1.39	252.04 ± 53.04	1194.27 ± 161.53	3.43 ± 0.17
Methyl ellagic acid I	2.45 ± 0.35	0.67 ± 0.26	431.06 ± 134.73	2128.94 ± 198.16	5.70 ± 0.31
Dimethyl ellagic acid I	26.23 ± 5.40	2.58 ± 1.63	176.74 ± 25.49	2648.96 ± 107.59	10.66 ± 0.28
Dimethyl ellagic acid IV	10.42 ± 2.81	2.58 ± 1.63	1232.82 ± 190.04	12,937.71 ± 1102.09	8.48 ± 0.62
Methyl ellagic acid sulfate II	4.52 ± 3.83	0.75 ± 0.61	59.08 ± 30.45	99.35 ± 27.69	1.70 ± 0.32
Dimethyl ellagic acid sulfate III	2.51 ± 0.90	1.33 ± 1.44	61.09 ± 15.64	301.85 ± 63.87	4.33 ± 0.49
Dimethyl ellagic acid glucuronide II	27.80 ± 21.29	5.33 ± 2.07	2005.41 ± 210.57	29,395.83 ± 1936.16	10.56 ± 0.45
Methyl gallic acid II	–	2.42 ± 1.36	13.47 ± 7.39	40.23 ± 17.59	2.71 ± 0.49
Gallic acid sulfate II	–	1.00 ± 0.77	5.34 ± 3.69	7.73 ± 2.81	1.65 ± 0.72
Methyl gallic acid sulfate I	1.58 ± 0.38	1.08 ± 0.74	220.32 ± 93.80	646.36 ± 140.82	2.72 ± 0.28
Dimethyl gallic acid sulfate I	–	1.08 ± 0.74	149.64 ± 31.06	398.43 ± 68.00	2.10 ± 0.14
Gallic acid glucuronide I	–	1.42 ± 0.66	23.52 ± 12.54	39.03 ± 15.70	1.65 ± 0.36
Methyl gallic acid glucuronide I	–	2.25 ± 1.47	13.00 ± 3.82	27.07 ± 9.40	2.01 ± 0.45
Urolithin A glucuronide	–	16.00 ± 6.20	167.85 ± 46.77	2108.55 ± 679.84	15.34 ± 1.46
Urolithin A sulfate	–	18.00 ± 6.57	9.82 ± 6.93	119.70 ± 75.32	16.65 ± 1.55
Urolithin A glucuronide and sulfate	–	20.00 ± 6.20	1.31 ± 1.42	12.78 ± 13.71	18.36 ± 2.36
Urolithin C glucuronide I	–	12.00 ± 6.20	11.97 ± 3.84	121.90 ± 20.85	13.27 ± 1.63
Urolithin C diglucuronide I	–	24.00 ± 0.00	4.78 ± 3.71	35.17 ± 22.13	21.24 ± 2.07
Methyl urolithin C glucuronide I	–	18.00 ± 6.57	1.25 ± 0.36	13.52 ± 3.66	16.55 ± 2.23
Methyl urolithin D glucuronide	–	11.33 ± 6.41	0.80 ± 0.50	5.24 ± 5.59	10.33 ± 3.90
Dimethyl urolithin D sulfate II	9.73 ± 4.10	1.42 ± 0.66	2.75 ± 0.55	10.08 ± 0.92	5.48 ± 1.36

The most abundant gallic acid conjugates were methyl gallic acid sulfate I (C_max_ = 220.32 ng/mL, AUC_0__–__24_ = 646.36 h·ng/mL), followed by dimethyl gallic acid sulfate I (C_max_ = 149.64 ng/mL, AUC_0__–__24_ = 398.43 h·ng/mL). Methyl gallic acid sulfate I and dimethyl gallic acid sulfate I were rapidly absorbed (T_max_ = 1.08 h), with MRT_0__–__24_ of 2.72 and 2.10 h, respectively. The remaining gallic acid conjugates demonstrated low systemic exposure and rapid clearance. Methyl gallic acid II, gallic acid sulfate II, gallic acid glucuronide I, and methyl gallic acid glucuronide I showed C_max_ values of 13.47, 5.34, 23.52, and 13.00 ng/mL, with corresponding AUC_0__–__24_ values of 40.23, 7.73, 39.03, and 27.07 h·ng/mL and MRT_0__–__24_ values of 2.71, 1.65, 1.65, and 2.01 h, respectively. Furthermore, all gallic acid conjugates fell below the detection limit within 8 h post-administration. Collectively, these results indicate that gallic acid conjugates are characterized by low systemic exposure and rapid absorption, although they exhibit short *in vivo* residence times.

Among urolithin conjugates, urolithin A glucuronide had the highest C_max_ (167.85 ng/mL) and AUC_0__–__24_ (2108.55 h·ng/mL), followed by urolithin C glucuronide I (C_max_ = 11.97 ng/mL, AUC0–24 = 121.90 h·ng/mL) and urolithin A sulfate (C_max_ = 9.82 ng/mL, AUC_0__–__24_ = 119.70 h·ng/mL). The majority of urolithin conjugates were detected after 4 h, exhibiting a delayed T_max_ (11.33–24 h) and prolonged MRT_0__–__24_ (10.33–21.24 h). A notable exception was dimethyl urolithin D sulfate II, which was detectable from 0.5 h with markedly earlier T_max_ (1.42 h) and a significantly shorter MRT_0__–__24_ (5.48 h). Additionally, several urolithin conjugates – urolithin A glucuronide, urolithin A sulfate, urolithin A glucuronide and sulfate, urolithin C glucuronide I, urolithin C diglucuronide I, and methyl urolithin C glucuronide I − maintained relatively high serum concentration levels at 24 h. The results indicate that urolithin conjugates are gradually produced after the metabolism of ellagitannins by gut microbiota, with relatively slow absorption rates and long *in vivo* residence times.

### 3.4 Proposed metabolic pathways of RPPs

**Fig. 2:**
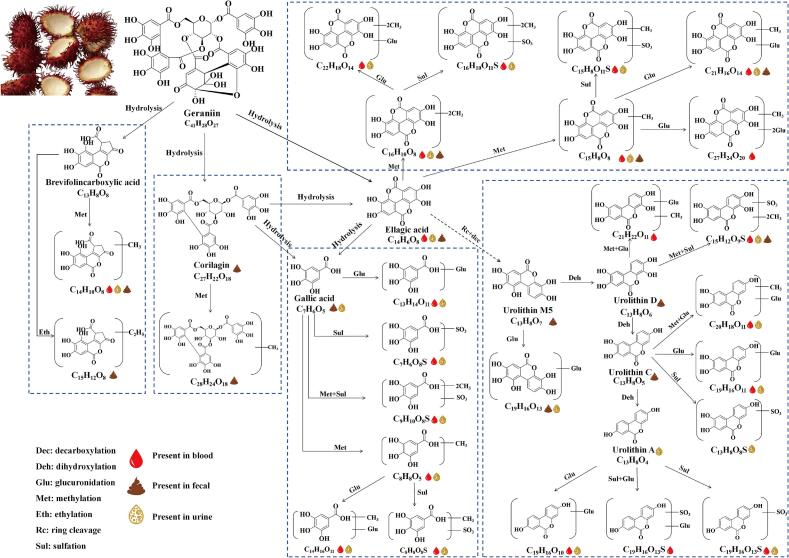
Metabolic pathways of rambutan peel polyphenols in rats.

The proposed metabolic pathways of RPPs in rats are illustrated in Fig. 2. Upon gastrointestinal digestion, geraniin undergoes hydrolysis influenced by physiological pH and enzymes throughout the digestive tract. This process liberates several phenolic compounds, including corilagin, brevifolincarboxylic acid, ellagic acid, and gallic acid, in varying amounts. The released compounds are further metabolized *via* phase II reactions (methylation, glucuronidation, sulfation), ultimately entering the bloodstream or being excreted as conjugates. Free ellagic acid enters the systemic circulation in the small intestine. Corilagin was not detected in the bloodstream. Instead, its prototype and hydrolyzed/methylated products were identified in feces, confirming that the majority of corilagin is not absorbed in the small intestine but instead passes into the colon. In the colon, corilagin can be hydrolyzed by microbiota, generating compounds like ellagic acid and gallic acid. Ellagic acid is further metabolized by gut microbiota (*via* ring cleavage and decarboxylation) into urolithin M5, which is then sequentially dehydroxylated to form urolithin D, C, and A. Urolithin M5 and D, along with their conjugates, are excreted in feces and urine. Urolithin C, and A undergo phase II metabolic reactions, entering systemic circulation or being excreted in the urine.

## 4 Discussion

Rambutan peel is an outstanding source of polyphenol. The main components of RPPs in this study are consistent with findings from several other studies (Li et al., 2025; Liu, Ma, et al., 2025; Zhuang et al., 2017a). These compounds include phenolic acids, flavonoids (flavonols, flavanols, flavones), ellagic acid and its derivatives, and hydrolyzable tannins, indicating the complexity of its chemical composition. RPPs in this study were rich in ellagitannins, particularly geraniin (341.24 mg/g). This aligns with the results of Phuong et al., who reported geraniin content as high as 397 mg/g extract in rambutan peel extracts (Phuong, Le, Dang, Van Camp, & Raes, 2020). Additionally, ellagic acid, ellagic acid pentoside II, and corilagin are also key characteristic components of RPPs. An increasing number of studies have revealed that RPPs possess significant biological activities (Hernández-Hernández et al., 2019). However, RPPs and their main components, geraniin and corilagin, exhibit very poor permeability across the Caco-2 cell monolayer, implying low bioavailability (Elendran, Muniyandy, Lee, & Palanisamy, 2019; Tan et al., 2023). Despite the low absorption of RPPs prototype components, they exhibit significant *in vivo* efficacy, strongly implicating their metabolites as the primary contributors to the observed biological effects.

In this study, the oral dose of RPPs administered to rats was 600 mg/kg BW. RPPs were rapidly and extensively transformed in rats, generating a complex mixture of phase II metabolites (methylated, glucuronidated, and sulfated derivatives) for systemic distribution and excretion. These conjugation reactions significantly enhance the solubility of the products in plasma and facilitate their excretion *via* urine. The detection of specific RPPs prototypes (*e.g.*, ellagic acid, ellagic acid pentoside II, and ellagic acid hexuronide) in serum confirms their direct absorption from the intestine. Despite this, these compounds exhibited limited systemic exposure and a short mean residence time compared to the various metabolites. Previous study reported that free ellagic acid was present in plasma at very low concentrations, but trace amounts of ellagic acid were also found in rat brain tissue, suggesting it may possess some tissue penetration ability (Ávila-Gálvez et al., 2025). The study by Chang et al. also detected ellagic acid pentoside in the serum and liver of tumor-bearing mice orally administered with *Phyllanthus emblica* L. extract, further confirming the *in vivo* distribution of such prototype components (Chang et al., 2025). We observed that ellagic acid reached its peak plasma concentration (C_max_ = 145.55 ng/mL) as early as 0.75 h after RPPs intake, but its concentration fell below the quantification limit after 12 h. In rats orally administered 0.8 g/kg pomegranate leaf extract, the T_max_ for ellagic acid in plasma was 0.55 h, and C_max_ was 213 ng/mL (Lei et al., 2003). In healthy humans after a single dose of 800 mg pomegranate extract, the C_max_ for ellagic acid in plasma was 33 ng/mL with T_max_ was 1 h (Mertens-Talcott, Jilma-Stohlawetz, Rios, Hingorani, & Derendorf, 2006). Following oral administration of pomegranate juice containing 318 mg punicalagins and 25 mg ellagic acid, the T_max_ for ellagic acid was 1 h, C_max_ was 31.9 ng/mL, and it was rapidly eliminated within 4 h (Seeram, Lee, & Heber, 2004). These results collectively indicate that the pharmacokinetic characteristics of ellagic acid are rapid absorption, distribution, and elimination. Furthermore, most ellagic acid and gallic acid conjugates peaked within 2 h RPPs intake, suggesting their primary site of absorption is small intestine.

The most abundant and long-lasting compounds in rat serum in this study were a series of methylated and glucuronidated ellagic acid derivatives. Dimethyl ellagic acid glucuronide II was particularly prominent, with its C_max_ (2005.41 ng/mL), AUC_0__–__24_ (29,395.83 h·ng/mL), and MRT_0__–__24_ (10.56 h) being 13.78, 51.84, and 2.99 times that of ellagic acid, respectively. As demonstrated in previous studies, ellagic acid derived from dietary ellagitannins is predominantly metabolized into methylated and glucuronidated forms in rats (Ma et al., 2016), and dimethyl ellagic acid glucuronide is a major plasma metabolite (Milala et al., 2017). Based on its high exposure and long residence time in circulation, dimethyl ellagic acid glucuronide II is likely a key circulating substance responsible for the *in vivo* effects of RPPs. Other conjugated forms of ellagic acid and gallic acid, due to their shorter MRT_0__–__24_ and lower systemic exposure, indicate they are rapidly absorbed and metabolized *in vivo*. Gallic acid is a common hydrolysis product of ellagitannins and one of the main components in RPPs. However, it was not detected in the serum in this study, possibly because the produced gallic acid concentration was low and it was rapidly metabolized and eliminated from the body (Lu et al., 2023). Previous study reported that after oral intake of ellagitannins, gallic acid and ellagic acid are detected *in vivo*, but they rapidly reach peak plasma concentrations and are quickly metabolized and excreted (Buyse et al., 2024; Ikeda et al., 2024). The gallic acid detected in feces might be a degradation product of geraniin or galloyl glucoses.

This study also identified hydrolysis and methylation products of corilagin. Study by Yisimayili et al. found that, after oral administration of pure corilagin, its metabolic pathways in rats included hydrolysis, methylation, glycosylation, reduction, glucuronidation, and sulfation, with ellagic acid and gallic acid being its characteristic metabolites (Yisimayili et al., 2019). This study found no evidence of corilagin entering the systemic circulation. Ellagic acid, derived from corilagin metabolites and RPPs prototypes, is gradually degraded by gut microbiota into a series of urolithin products (Gandhi et al., 2022). In our study, a complete urolithin metabolic profile (from urolithin M5, D, C to A, and their various conjugates) was identified in serum, urine, and feces. These substances are products of the deep microbial metabolism in the colon of unabsorbed ellagitannins (*e.g.*, geraniin and corilagin) and ellagic acid. Ikeda et al. found that after intake of an ellagitannin-rich extract, the time to maximum concentration for urolithin products (urolithin A, C, M3, M4) in rat plasma was 20–36 h (Ikeda et al., 2024). In our study, most urolithin conjugates became detectable in rat serum after 4 h of RPPs administration, with T_max_ values exceeding 10 h. It can be seen that urolithin metabolites exhibited a delayed systemic appearance compared to the RPPs prototypes (*e.g.*, ellagic acid, ellagic acid pentoside II, and ellagic acid hexuronide). Following intestinal absorption, these urolithins subsequently underwent further phase II metabolism, including glucuronidation and sulfation, while sulfate forms have much lower concentrations than glucuronide forms (Ares et al., 2023; Milala et al., 2017; Singh et al., 2021). Our results are consistent with these findings, as the serum level of urolithin A glucuronide was much higher than other urolithin conjugates, and it was the main circulating substance detected in the pharmacokinetic analysis. Studies have found that from tetrahydroxylated urolithin to monohydroxylated urolithin, their lipophilicity and absorption rate tend to increase (Luca et al., 2019).

Absorption of geraniin was found to be negligible, as evidenced by its absence in the serum, urine, and feces of rats. On the one hand, the high polarity of geraniin itself limits its solubility in gastric juice and its permeability through the intestinal lipid membrane (Elendran, Wang, Prankerd, & Palanisamy, 2015). On the other hand, upon entering the body, geraniin undergoes hydrolysis in the gastrointestinal tract due to physiological conditions, partially degrading into smaller molecules (corilagin, ellagic acid, gallic acid) (Tan et al., 2023). Upon absorption from the small intestine, a portion of the ellagic acid enters the bloodstream and is transported to the liver, where it undergoes phase II reactions that enhance its solubility and stability. The geraniin, corilagin, and ellagic acid not absorbed in the small intestine continue into the colon, where they are hydrolyzed to varying degrees by gut microbiota, ultimately generating a series of urolithin products. Gut microbiota-derived urolithin A is extensively metabolized in the liver to urolithin A glucuronide before entering systemic circulation (Scott, Styring, & McCullagh, 2022). Subsequently, this metabolite undergoes deglucuronidation mediated by β-glucuronidase, releasing the urolithin A aglycone again (Bobowska et al., 2020). Urolithin A has been widely studied and found to possess biological activities such as antioxidant, anti-inflammatory, anticancer, anti-obesity, and anti-aging effects (Zhang et al., 2022). By converting high-molecular-weight ellagitannins into low-molecular-weight urolithins, the gut microbiota significantly enhances the bioavailability and biological activity of RPPs. However, the metabolic process of ellagitannins and the resulting bioactivity of urolithins show significant inter-individual variation. Current research reports four urolithin metabotypes (urolithin metabotype A, B, 0, and MR), and different metabotypes affect the types of final urolithin products, which is also an important factor leading to variations in polyphenol biological effects (Liu, Sun, et al., 2025). Furthermore, while the health effects of polyphenolic compounds (*e.g.*, ellagic acid, urolithins) have been widely confirmed by research, the biological activity performance of conjugated forms of polyphenols after *in vivo* metabolism still needs further investigation.

## 5 Conclusion

In summary, this study systematically elucidates the *in vivo* fate of RPPs following oral administration. RPPs exert their biological activity, rather than solely *via* the parent compounds, but also through two key classes of metabolites produced by host and gut microbiota metabolic processes. 1) Host-mediated rapid response pathway. Ellagic acid derivatives, represented by dimethyl ellagic acid glucuronide, exhibit high exposure and long residence time, constituting a direct, rapid, and long-lasting active carrier. 2) The microbiota-host co-metabolism delayed-persistence pathway. This pathway is characterized by urolithin metabolites, which exhibit a delayed appearance but a prolonged *in vivo* duration, thereby constituting a pool of bioactive compounds with sustained effects. These two classes of metabolites are kinetically complementary and potentially synergistic in function, together constituting the complete *in vivo* material basis and pharmacodynamic foundation of rambutan peel polyphenols after oral administration. Although a well-established LC-MS method was used, the lack of authentic standards limits the results to semi-quantitative analysis. Moreover, the short-term single-gavage design did not allow assessment of the interactions between RPPs and the gut microbiota. Future studies should obtain key standards for absolute quantification and perform long-term experiments to identify the specific microbial populations responsible for RPPs metabolism, thereby fully elucidating the host–microbiota interactions.

## CRediT authorship contribution statement

**Qiuming Liu:** Writing – original draft, Methodology, Formal analysis. **Wanmei Biao:** Methodology, Investigation. **Xingchun Duan:** Methodology, Investigation. **Yujie Zhong:** Software, Data curation. **Qingyu Ma:** Methodology, Conceptualization. **Liping Sun:** Writing – review & editing, Supervision. **Yongliang Zhuang:** Writing – review & editing, Visualization, Supervision, Data curation.

## Declaration of competing interest

The authors declare that they have no known competing financial interests or personal relationships that could have appeared to influence the work reported in this paper.

## Data Availability

Data will be made available on request.
